# Benzoxazinoids in Root Exudates of Maize Attract *Pseudomonas putida* to the Rhizosphere

**DOI:** 10.1371/journal.pone.0035498

**Published:** 2012-04-24

**Authors:** Andrew L. Neal, Shakoor Ahmad, Ruth Gordon-Weeks, Jurriaan Ton

**Affiliations:** 1 Centre for Sustainable Soils and Grassland Systems, Rothamsted Research, Harpenden, Hertfordshire, United Kingdom; 2 Institute of Environmental Biology, Utrecht University, Utrecht, The Netherlands; 3 Department of Animal and Plant Sciences, University of Sheffield, South Yorkshire, United Kingdom; University of Wisconsin-Milwaukee, United States of America

## Abstract

Benzoxazinoids, such as 2,4-dihydroxy-7-methoxy-2*H*-1,4-benzoxazin-3(4*H*)-one (DIMBOA), are secondary metabolites in grasses. In addition to their function in plant defence against pests and diseases above-ground, benzoxazinoids (BXs) have also been implicated in defence below-ground, where they can exert allelochemical or antimicrobial activities. We have studied the impact of BXs on the interaction between maize and *Pseudomonas putida* KT2440, a competitive coloniser of the maize rhizosphere with plant-beneficial traits. Chromatographic analyses revealed that DIMBOA is the main BX compound in root exudates of maize. *In vitro* analysis of DIMBOA stability indicated that KT2440 tolerance of DIMBOA is based on metabolism-dependent breakdown of this BX compound. Transcriptome analysis of DIMBOA-exposed *P. putida* identified increased transcription of genes controlling benzoate catabolism and chemotaxis. Chemotaxis assays confirmed motility of *P. putida* towards DIMBOA. Moreover, colonisation essays in soil with *Green Fluorescent Protein* (*GFP*)-expressing *P. putida* showed that DIMBOA-producing roots of wild-type maize attract significantly higher numbers of *P. putida* cells than roots of the DIMBOA-deficient *bx1* mutant. Our results demonstrate a central role for DIMBOA as a below-ground semiochemical for recruitment of plant-beneficial rhizobacteria during the relatively young and vulnerable growth stages of maize.

## Introduction

Plants have evolved to interact with soil-borne microbes. In addition to arbuscular mycorrhizal fungi and nodule-forming rhizobia, plants interact with a wide range of rhizosphere-colonising bacteria. These are attracted to root surfaces by chemical components in root exudates, which are rapidly assimilated into microbial biomass [1]. This so-called rhizosphere effect supports bacterial cell densities in the root vicinity up to 100-fold greater than in surrounding soil [2]. The chemical composition of root exudates differs between plant species and evidence suggests that the structure of bacterial communities in the rhizosphere differs accordingly [3]. Observations that the rhizosphere community is directly influenced by plant species have led to the hypothesis that plants may recruit specific bacteria [4]. However, it remains difficult to determine whether plants are actively recruiting specific microbes, or whether dominance of a limited number of bacterial species is simply based on a greater ‘fitness’ to exploit root exudates [5].

When rhizospheric dominance by a single micro-organism occurs, the plant-microbe interaction can range from deleterious, in the case of phytopathogens, to beneficial, where rhizobacteria can promote plant growth and resistance to plant stress. Growth promotion by rhizobacteria involves a variety of different mechanisms, including N_2_-fixation by diazotrophs [6] and improved availability of poorly soluble inorganic ions, such as PO_4_^3−^ and Fe[III], but can also result from modulation of plant regulatory mechanisms, such as phytohormone homeostasis [7]. In addition, rhizobacteria can promote growth indirectly by protecting the host plant against pests and diseases. This protection can be based on direct antibiosis or competition for nutrients [8], but can also result from induced systemic resistance (ISR) [9].

Evidence suggests that plant-associating bacteria have evolved the ability to metabolise plant-derived aromatic compounds [10]. For instance, plant-associating bacteria have been shown to metabolise umbelliferone, salicylic acid and 4-hydroxybenzoate [10]. As a consequence, these bacteria are often also capable of metabolising aromatic pollutants, such as naphthalene, toluene and 2,4-dichlorophenoxyacetic acid [10]. Some aromatic acids can also act as bacterial chemo-attractants [11], suggesting that plant derived aromatic compounds could serve to recruit plant-beneficial rhizobacteria to the rhizosphere.

Benzoxazinoids (BXs), such as 2,4-dihydroxy-7-methoxy-2*H*-1,4-benzoxazin-3(4*H*)-one (DIMBOA), are heteroaromatic metabolites with benzoic acid moieties [12]. Since their identification as major secondary defence metabolites in *Poaceae*, investigations have predominantly focussed on their role in plant defence against above-ground pests and pathogens [13], [14]. BXs are typically produced during relatively early, vulnerable plant growth stages [12]. In response to tissue damage, vacuolar reservoirs of BX-glucosides are hydrolysed by plastid-targeted β-glucosidases, causing rapid accumulation of aglucone BX biocidal metabolites [15]. A recent study in maize revealed that *Spodoptera* larvae can detoxify DIMBOA by glycosylation and that the contribution of maize BXs to defence against these herbivores is based on an inducible conversion of DIMBOA-glc into 2-β-d-glucopyranosyloxy-4,7-dimethoxy-1,4-benzoxazin-3-one (HDMBOA-glc) [16]. Interestingly, BXs are also active against attackers causing relatively minor tissue damage, such as aphids and pathogenic fungi. This function is based on an increased accumulation of DIMBOA in the apoplast before the onset of large-scale tissue damage, where it signals increased deposition of callose-rich papillae [17]. Thus, the above-ground defence contribution of BXs is not only limited to their biocidal properties, but also includes a within-plant signalling function in the activation of plant innate immune responses against pests and diseases.

BXs have also been implicated in plant defence below-ground. BXs are exuded in relatively large quantities from cereal roots, where they can act as allelochemicals against microbes, insects or competing plants [13], [18]. Once released, BXs degrade relatively quickly in aqueous environments with a half-life of less than 24 hours [19]. Upon hydrolysis, DIMBOA is converted into 6-methoxy-benzoxazolin-2-one (MBOA), a compound considerably more stable in sterile soil, but with significantly less toxicity than DIMBOA [20]. Biodegradation of MBOA leads to accumulation of phenoxazinones [21], and requires activity by microbes, such as *Acinetobacter calcoaceticus* [22], soil-borne fungi [23], or unidentified members in the rhizosphere community of oat [24]. Phenoxazinone products are typically more biocidal than benzoxazolinones and have antifungal [25], antibacterial [26], and plant allelopathic properties [27]. Hence, BX exudation by plants can have a major impact on rhizosphere communities in the soil.

Plant-derived aromatic metabolites can act as chemo-attractants for *Pseudomonas putida* [10], [11]. We therefore hypothesised that BXs from root exudates of maize may attract and support *P. putida* cells. To address this hypothesis, we studied the influence of BXs on *P. putida* KT2440, a competitive coloniser of the maize rhizosphere with plant-beneficial traits [28], [29]. We identified DIMBOA as the dominant BX species in maize root exudates and found that exposure of *P. putida* to DIMBOA induces bacterial genes with putative functions in chemotactic responses. *In vitro* chemotaxis assays indeed revealed that *P. putida* KT2440 displays taxis towards DIMBOA. The ecological relevance of this response was confirmed by root colonisation assays in soil, using maize mutant lines impaired in BX biosynthesis. Our study presents evidence that root exudation of DIMBOA during the vulnerable growth stages of maize promotes colonization by plant-beneficial rhizosphere bacteria.

## Materials and Methods

### Plant material and cultivation

Maize lines were derived from reciprocal crosses between a *bx1* single mutant and an indole-deficient *igl* mutant, as described by Ahmad *et al.* [17]. Since the *bx1* single mutant contains residual levels of benzoxazinoids due to a functional *Indole-3-Glycerol phosphate Lyase (IGL)* gene [17], comparisons within each progeny were made between the benzoxazinoid-producing *BX1* and benzoxazinoid-deficient *bx1* genotypes in the background of the *igl* mutant genotype (i.e. *BX1 igl* versus *bx1 igl*). For each experiment, progenies from two independent crosses (Line A and Line B) were analysed for phenotypes. Seeds were allowed to germinate at 22°C and high humidity in petri-dishes in the dark. Germinated seedlings of similar size were planted in pots containing compost and were cultivated under controlled conditions (16:8 h L:D, 22°C).

### Bacterial strains and cultivation

Two *Pseudomonas putida* strains were used. KT2440 was used for all *in vitro* experiments, including transcriptome profiling. For soil experiments, a green fluorescent protein (GFP)-tagged KT2440 derivative strain, FBC004, was used which carries a stable chromosome-inserted PA_1/04/03_-RBSII-*gfp*mut3*-T0-T1 transposon at a negligible metabolic cost [30]. Stocks of KT2440 and FBC004 were routinely stored at −80°C. For each experiment, fresh cultures were started from stocks. Depending on the experiment, cells were grown overnight at 21°C with 150 r.p.m. agitation, either in LB medium, or in M9 minimal medium supplemented with 0.1% glucose as the sole carbon source. To assess tolerance of *P. putida* to DIMBOA, the ubiquitous soil bacterium *Agrobacterium tumefaciens* was used as a comparator. *A. tumefaciens* was grown in M9 medium supplemented with 0.1 µM FeCl_3_. In this case, growth of the two bacteria was followed by assessing OD_600_ in five replicate 200 µL cultures at 21°C in 96-well plates with a Varioskan plate reader (Thermo Scientific, Cramlington, UK)

### *Pseudomonas putida* transcriptome response to DIMBOA

To test the response of *P. putida* to DIMBOA, we employed a KT2440 specific cDNA microarray [31], [32]. Preliminary experiments indicated that DIMBOA hydrolyses rapidly in M9 medium (half-life, 21 hours). Therefore, to test the bacterial response to DIMBOA, *P. putida* KT2440 cells were grown to mid-exponential phase in 100 mL M9 medium before DIMBOA was added to a final concentration of 5 µg mL^−1^. After 1 hour of exposure, cells were harvested by centrifugation at 4°C. RNA was extracted from three independently performed experiments. Cell pellets were treated with RNAprotect™ (Qiagen, Valencia CA) immediately following centrifugation. Cell membrane lysis was achieved with 1 mg mL^−1^ lysozyme in buffer containing 10 mM TRIS and 1 mM EDTA at pH8 using Qiagen RNeasy® reagent kits following the manufacturer's instructions. Extracted RNA was purified with TURBO DNA-*free*™ kits (Ambion, Applied Biosystems, Foster City, CA) and quantified on a NanoDrop 1000 spectrophotometer. cDNA was synthesised and labelled using the SuperScript™ indirect cDNA labelling system (Invitrogen, Carlsbad, CA). Synthesised cDNA paired samples (control or DIMBOA) were labelled with Cy3 or HyPer5 (Amersham, Little Chalfont, UK) fluorophores. To remove dye bias, the experimental design included dye-swap normalisation procedures, as described by Dabney & Storey [33]. Dye incorporation was verified to be more than 150 pmol dye per sample. Equal amounts of Cy3-cDNA and HyPer5-cDNA, each representing a replicate comparison between control and DIMBOA-treated cells, were combined and dried in a speedvac before proceeding with array hybridisation. Microarrays were pre-treated with BlockIt™ Plus blocking buffer (Arrayit Corporation, Sunnyvale, CA) in order to inactivate reactive groups on the surface. Dried cDNA was rehydrated in buffer and hybridised to arrays (Progenika Biopharma S.A, Vizcaya, Spain) for 18 hours at 42°C, according to the manufacturer's instructions. Following hybridisation, arrays were washed, dried, and scanned with a GenePix® 4000B scanner (Molecular Devices, Sunnyvale, CA). Data were processed using TM4 microarray software [34]. Data from the three independent replicate experiments were combined and analysed together. Using Statistical Analysis for Microarrays procedures [35], only genes that were consistently induced by DIMBOA in all three independent replicates were considered significant. Functional annotation of induced genes was performed using the supporting microarray documentation and the *P. putida* KT2440 KEGG genome database (www.genome.jp/kegg-bin/show_organism?org=ppu).

### *In vitro Pseudomonas putida* KT2440 chemotaxis assay

Chemo-attractiveness of DIMBOA was quantified using a modified capillary-based chemotaxis assay [11], which relies on accumulation of bacterial cells in microcapillary tubes (1 µL volume, Drummond Scientific Company, Broomall, PA). Tubes containing glucose-free M9 medium (control), M9 medium with 5 µg g^−1^ DIMBOA, or M9 medium with 0.1% casamino acids (positive control) [11] were incubated in individual wells of a 96-well plate. Each well contained 200 µL glucose-free M9 suspension with *P. putida* KT2440 bacteria (OD_600_ = 0.06). After 30 minutes, capillary contents were carefully collected and plated onto LB agar for cell enumeration.

### Maize - *Pseudomonas putida* FBC004 colonisation assays

Seeds germinating after 2 days of imbibition were planted in 100 mL-pots (3 seeds per pot; 4 pots per genotype), containing autoclaved (120°C; 20 min) or non-autoclaved soil that had been supplemented with washed cells from overnight FBC004 cultures at an approximate density of 5×10^7^ colony forming units (CFU) g^−1^ soil. At 7, 14, and 21 days of growth, root systems were gently removed from the soil, rinsed in water, weighed and gently shaken for 20 minutes in 50 mL phosphate-buffered saline (mmol L^−1^; NaCl 137, KCl 2.7, Na_2_HPO_4_·2H_2_O 10, KH_2_PO_4_ 1.76; pH 7.4). Serial dilutions of rhizosphere bacteria were plated onto LB agar, containing 200 mg L^−1^ cyclohexamide to inhibit fungal growth. Plates were incubated at room temperature for 48 hours. GFP-expressing colonies were counted using a DR88X Dark Reader® transilluminator (Clare Chemical Research Inc., Dolores, CO); the total numbers of non-*P. putida* (other) culturable cells were determined under natural light. Root colonisation by *P. putida* and other culturable cells, assessed as CFU g^−1^ root fresh weight, was analysed for each maize line and time point by two-factor analysis of variance (ANOVA), using the maize BX genotype (*BX1*, *bx1*) and bacterial cell type (*P. putida*, other culturable cells) as factors. All data were log_10_-transformed before analysis to stabilise variances. Post-test comparisons were made using Holm-Šidák step-down pairwise comparisons. All statistical analyses were performed using SigmaPlot version 12.

### Extraction and chromatographic analysis of benzoxazinoids in root exudates and bacterial cultures

Seeds germinating after 2 days imbibition were planted in soil (3 seeds per pot). At days 7, 14, and 21 after planting, root systems were gently removed from the soil, rinsed in water, and placed in 50 mL tubes containing 30 mL water for seven hours to collect root exudates. Root exudates were lyophilised, re-suspended in 1 mL extraction buffer (2% acetic acid in methanol), sonicated for 5 min and centrifuged (12,600× *g*, 10 min). Supernatants were analysed by high performance liquid chromatography coupled to diode array detection (HPLC-DAD), as described by Ahmad *et al.* [17]. Root exudates from *BX1* genotypes contained three main peaks, absent in samples from *bx1* genotypes. Spiking experiments with previously confirmed standards [16], [17] revealed that exudates from *BX1* wild-type roots consistently contain three main BX species: DIMBOA and to a lesser extent DIMBOA-glc and HDMBOA-glc. For analysis of DIMBOA breakdown by *P. putida* KT2440 in M9 growth medium, 0.5 mL samples were periodically removed and filtered (<0.2 µm) to remove cells. The samples were then stored in an equal volume of extraction buffer until analysis. Detection of DIMBOA and MBOA was based on a modified HPLC protocol, using a mobile phase of 0.05% trifluoroacetic acid in water (solution A) and 0.05% trifluoroacetic acid in methanol (solution B) at a flow rate of 1 mL min^−1^. The gradient consisted of 0–1 minute 3–20% solution B, 1–20 minutes, 20–100% solution B, and 20–35 minutes isocratic conditions of 100% solution B. Chromatograms were recorded at 254 nm and retention times of DIMBOA and MBOA were established from standards.

## Results

### Exudation of benzoxazinones from maize roots

**Figure 1:**
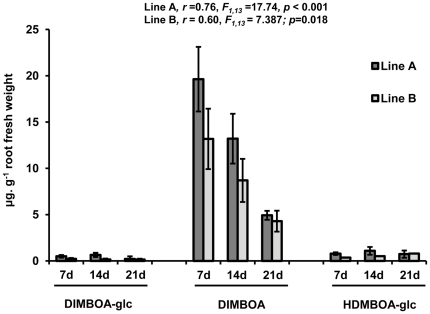
Root exudation of benzoxazinoids at different developmental stages in maize lines expressing a functional *BX1* gene. The dominant BX compound in root exudates is the aglucone 2,4-dihydroxy-7-methoxy-1,4-benzoxazin-3-one (DIMBOA), which shows a statistically significant linear decrease with plant age in both lines. Shown are average BX quantities, expressed as µg g^−1^ root fresh weight (± SEM; *n* = 3) exuded over a 7 hour time period.

Roots of *BX1* wild-type and *bx1* mutant lines were incubated for 7 hours in water, after which the collected exudates were subjected to HPLC-DAD analysis of BXs. Root exudates from *BX1* wild-type plants consistently contained three BX compounds, all of which were absent from exudates of *bx1* mutant lines. The dominant compound was DIMBOA, with concentrations up to 31 µg g^−1^ fresh root weight (FW) in exudates from 7 days old roots (**Figure 1**). Levels of DIMBOA exudation showed a statistically significant linear decline in aging plants (**Figure 1**; linear regression, Line A, *F*_1,13_ = 17.74; *p*<0.001, Line B, *F*_1,13_ = 7.387; *p* = 0.018). The other plant-derived BXs in root exudates from *BX1* expressing plants were the BX glucosides DIMBOA-glc and HDMBOA-glc. Concentrations of these compounds did not exceed 3 µg g^−1^ FW and remained constant over time.

### *P. putida* is tolerant to DIMBOA

**Figure 2:**
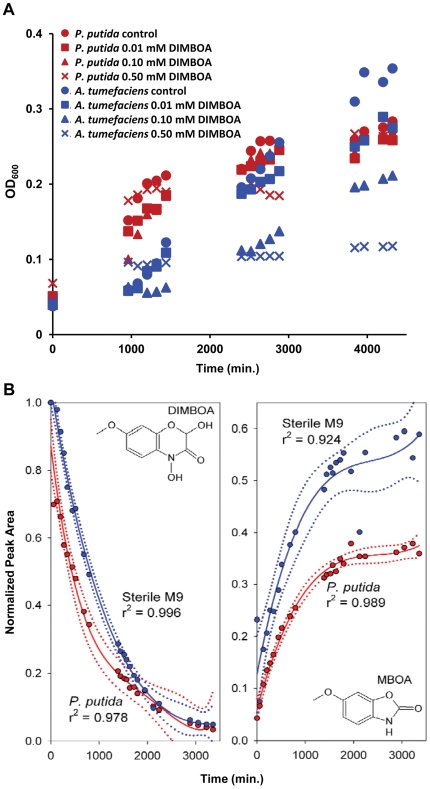
Tolerance of *Pseudomonas putida* KT2440 to DIMBOA. **A.** *In vitro* growth of *P. putida* KT2440 is not affected by up to 0.5 mM DIMBOA, whereas the ubiquitous soil bacterium *Agrobacterium tumefaciens* is increasingly affected at concentrations of 0.01 mM DIMBOA and greater. Growth was quantified by determining average OD_600_ values (*n* = 5). **B.** In the presence of *P. putida* KT2440, DIMBOA degradation is significantly accelerated, but accumulation of MBOA is significantly reduced. Shown are best fitting polynomial regressions ±99% confidence intervals. DIMBOA and MBOA quantities are expressed as relative peak areas (HPLC-DAD), normalised to DIMBOA peak areas at the start of each experiment.

To examine the effect of DIMBOA on plant-beneficial rhizobacterial growth, we assessed *in vitro* growth of *P. putida* KT2440 bacteria in the presence of increasing concentrations of DIMBOA. *P. putida* KT2440 displayed similar growth rates up to 0.5 mM DIMBOA. By contrast, DIMBOA strongly affected growth rates of the ubiquitous soil bacterium *Agrobacterium tumefaciens* [36], effects were already apparent at 0.01 mM and became proportionally more pronounced at 0.1 and 0.5 mM DIMBOA (**Figure 2A**). Hence, *P. putida* KT2440 appears relatively tolerant to DIMBOA in comparison to other soil bacteria. For all subsequent experiments, DIMBOA was employed at concentrations of 5 µg mL^−1^ (0.023 mM). This relatively low concentration has no detrimental effect on *P. putida* growth (**Figure 2A**) and is quantitatively consistent with our root exudation experiments (**Figure 1**).

### *P. putida* accelerates DIMBOA breakdown

To study whether the observed tolerance of *P. putida* KT2440 to DIMBOA is based on BX catabolism, we studied the effect of *P. putida* KT2440 on stability of DIMBOA and its direct break-down product, 6-methoxy-benzoxazolin-2-one (MBOA). In two independent experiments, DIMBOA concentrations were consistently reduced at a significantly greater rate, whereas MBOA accumulation was significantly reduced in the presence of *P. putida* bacteria (**Figure 2B**). These results demonstrate that *P. putida* KT2440 accelerates breakdown of DIMBOA. The reduced accumulation of MBOA in the presence of *P. putida* could be explained by a more rapid metabolic break-down of this compound, but could also suggest DIMBOA degradation via products other than MBOA. Although is not possible to distinguish which of these processes is responsible for the observed compound dynamics in the presence of *P. putida*, our results clearly show that *P. putida* has the metabolic capacity to degrade DIMBOA and reduce overall BXs quantities in its environment.

### Impact of DIMBOA on the *P. putida* transcriptome

**Figure 3:**
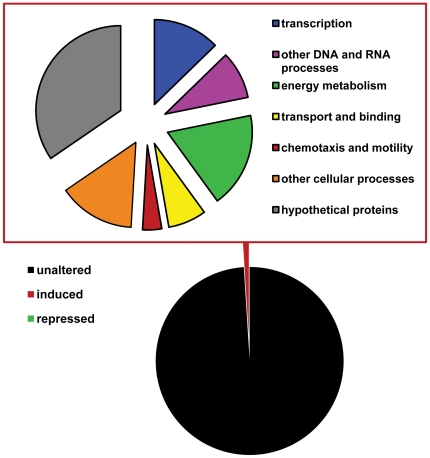
Functional annotation of 55 DIMBOA-inducible genes of *P. putida* KT2440 at 1 hour after exposure to 5 µg mL^−1^ DIMBOA in the growth medium. Whole-genome transcriptome analysis was based on *P. putida* KT2440-specific cDNA microarrays, accommodating results from three independent experiments.

The above *in vitro* analyses suggest that DIMBOA is metabolised by *P. putida*. To assess the global impact of DIMBOA on *P. putida* KT2440, whole-genome gene expression patterns were profiled at 1 hour of exposure to 5 µg mL^−1^ DIMBOA in M9 growth medium, deposited in NCBI's Gene Expression Omnibus (GSE36489). Using KT2440-specific cDNA microarrays and a false discovery rate of 0.85% (Δ = 1.2) [35], we identified 55 genes showing consistently increased levels of transcription in response to DIMBOA treatment across three independent experiments. No genes were identified as significantly repressed by DIMBOA. A total of 36 genes could be ascribed predicted functions, whereas 19 genes encoded hypothetical proteins of unknown function (**Figure 3**). The 36 DIMBOA-inducible genes with identifiable function are further detailed in **Table S1**. Two groups of genes were of particular interest with respect to *P. putida* behaviour in the rhizosphere. One group of genes are typical of those associated with degradation of *N*-heteroaromatic compounds (**Table S1**), and are consistent with the accelerated breakdown of DIMBOA by *P. putida* KT2440 (**Figure 2B**). A second group of genes are indicative of bacterial motility (**Figure 3** **; Table S1**), thereby suggesting a chemotactic response of *P. putida* KT2440 to DIMBOA.

### DIMBOA induces positive chemotaxis by *P. putida*

**Figure 4:**
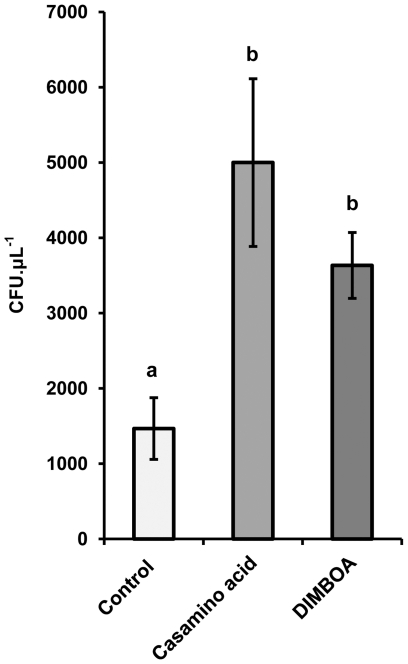
Taxis of *P. putida* KT2440 towards DIMBOA. A capillary-based assay was used to assess chemotactic responses. Data represent average numbers of colony forming units (CFU ± SEM) from 1 µL glass capillaries containing motility buffer (control), 0.1% casamino acid (positive control), or 5 µg mL^−1^ DIMBOA. Cells were extracted from capillaries after 30 minutes of incubation and enumerated on solid medium.

Based on the outcome of the transcriptome analysis, we examined the possibility that DIMBOA acts as a chemo-attractant for *P. putida* KT2440. A capillary-based assay was used to assess chemotactic behaviour to DIMBOA [11]. Significantly more cells (*p* = 0.022; *t*-test) were attracted into capillaries containing 5 µg mL^−1^ DIMBOA compared to tubes with motility buffer alone (**Figure 4**). The average number of DIMBOA-attracted cells was statistically similar to the average number of cells that were attracted to the positive control tubes, containing 0.1% w/v casamino acids (**Figure 4**). Hence, *P. putida* KT2440 is attracted to DIMBOA *in vitro*.

### DIMBOA attracts *P. putida* to the rhizosphere

Having established that DIMBOA induces chemotaxis-associated genes in *P. putida* KT2440, and that *P. putida* KT2440 is attracted to DIMBOA *in vitro*, we investigated whether these responses are biologically relevant in the maize rhizosphere. To this end, GFP-expressing cells of *P. putida* FBC004 were mixed into the soil prior to planting seeds of either DIMBOA-producing wild-type plants (*BX1*), or BX-deficient *bx1* mutant plants. After 7, 14 and 21 days of growth, roots of *BX1* and *bx1* plants from 2 independent genetic lines were collected and analysed for colonisation by *P. putida* FBC004 and other (non-GFP expressing) culturable rhizobacteria.

**Figure 5:**
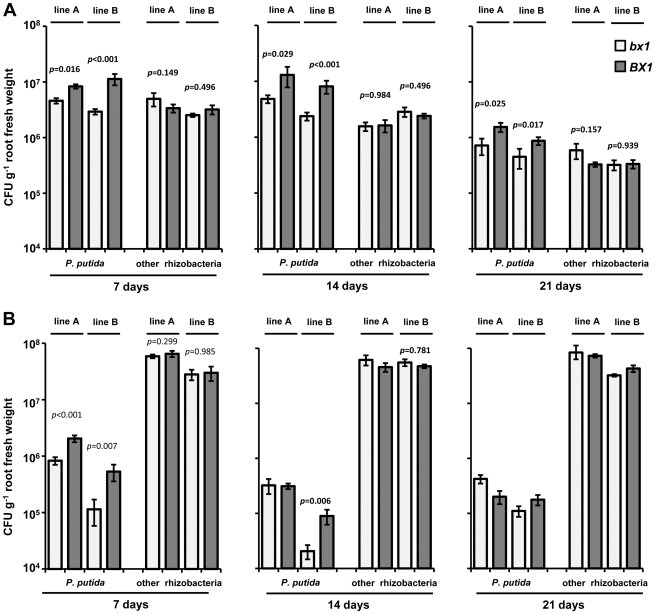
Rhizosphere colonisation of DIMBOA-producing (*BX1*) and DIMBOA-deficient (*bx1*) maize lines by green fluorescent protein (GFP)-expressing *P. putida* and other culturable rhizobacteria in autoclaved (**A**) and non-autoclaved soil (**B**). *P. putida* cells were introduced into the soil prior to planting of maize seeds. Shown are average values (CFU g^−1^ root fresh weight ± SEM; *n* = 6–8), corresponding to *P. putida* or other rhizobacteria. Cells were enumerated after 7, 14 and 21 days of plant growth by plating root surface washes onto solid agar medium. Probabilities indicate the likelihood of the differences between *BX1* and *bx1* plants, within one line at each time-point, occurring by chance (Holm-Šidák pair-wise multiple comparisons) when two-factor ANOVA indicated a statistically significant (α = 0.05) main effect of BX genotype and/or a statistically significant interaction between maize BX genotype and bacterial cell type.

The first experiment was performed with soil that had been autoclaved once before the start of the experiment (**Figure 5A**), presenting a relatively low competition environment for introduced *P. putida*. Two-factor ANOVA of rhizosphere colonisation of plants from Line A revealed a statistically significant interaction between plant genotype (*BX1 versus bx1*) and bacterial cell type (*P. putida versus* others) at all three time-points (7 days: *F*_1,14_ = 9.151, *p* = 0.009; 14 days: *F*_1,14_ = 43.432, *p*<0.001; 21 days: *F*_1,14_ = 7.977; *p* = 0.014), even though a statistically significant main effect of BX genotype could not be detected. Inspection of the data revealed that more *P. putida* cells were recovered from roots of DIMBOA-producing *BX1* plants than from roots of DIMBOA-deficient *bx1* plants; this was not the case for numbers of other culturable rhizobacteria. Holm-Šidák comparisons confirmed significantly higher *P. putida* cell numbers in rhizosphere washes from *BX1* roots compared to that from *bx1* roots (statistical probabilities are presented in **Figure 5A**). For line B, a significant main effect of BX genotype was evident at day 7 (*F*_1,16_ = 18.163; *p*<0.001) and day 14 (F_1,16_ = 19.776; *p*<0.001), but not at day 21 (*F*_1,16_ = 3.775; *p* = 0.070). However, a statistically significant interaction between BX genotype and rhizobacterial cell type was apparent at all three time-points, including day 21 (*F*_1,16_ = 6.122; *p* = 0.025). Again, Holm-Šidák comparisons indicated significantly greater numbers of *P. putida* cells in the rhizosphere of *BX1* plants compared to *bx1* plants at all three time points (**Figure 5A**). Hence, *BX1*-dependent exudation of DIMBOA stimulates rhizosphere colonisation by *P. putida* bacteria.

To investigate whether BX-dependent attraction of *P. putida* is also apparent in a more competitive soil environment, we repeated the experiment in non-autoclaved soil (**Figure 5B**). A significant main effect of BX genotype was observed at day 7 in seedlings from line A (*F*_1,10_ = 6.725; *p* = 0.027), but not at any later growth stage. Holm-Šidák comparisons confirmed significantly higher numbers of *P. putida* cells in the rhizopshere of 7-day-old *BX1* seedlings, but no statistically significant difference in the number of other culturable rhizobacteria (statistical probabilities are presented in **Figure 5B**). No main effect of BX genotype was identified for plants of line A at days 14 (*F*_1,11_ = 0.110; *p* = 0.746) or 21 (*F*_1,12_ = 4.152; *p* = 0.064). For line B, a significant main effect of BX genotype was observed at 7 days (*F*_1,11_ = 4.904; *p* = 0.049). Although no significant main effect of BX genotype was observed at 14 days (*F*_1,12_ = 4.547; *p* = 0.054), there was a significant interaction between BX genotype and rhizobacterial cell type at this time-point (*F*_1,12_ = 6.425; *p* = 0.026). Subsequent Holm-Šidák comparisons confirmed significantly increased numbers of *P. putida* in the rhizosphere of *BX1* plants at both 7 and 14 days (**Figure 5B**). At 21 days there was no longer a statistically significant main effect of plant genotype, nor was there a statistically significant interaction between plant genotype and rhizobacterial cell type (**Figure 5B**). Together, these data indicate that BX exudation in non-autoclaved soil stimulates rhizosphere colonisation by *P. putida* of relatively young seedlings. This BX effect becomes variable by 14 days and is absent in 21 day-old plants.

## Discussion

The rhizosphere is an energy-rich niche that is characterised by a rapid turnover of chemical compounds exuded from plant roots [37]. Before rhizobacteria can exploit these compounds in the rhizosphere, they must first locate their host and tolerate potentially toxic allelochemicals in root exudates. In this study, we provide evidence that rhizosphere-colonising *P. putida* cells are tolerant of the *N*-heteroaromatic allelochemical DIMBOA (**Figure 2**), which is exuded in relatively high quantities from roots of young maize seedlings (**Figure 1**). Since BXs are nitrogen-containing metabolites, it might be expected that constitutive DIMBOA exudation by seedlings must provide significant ecological benefits, outweighing the high metabolic cost. Apart from allelopathatic activity by DIMBOA [13], [14], our study revealed that DIMBOA also acts as a below-ground semiochemical for recruitment of plant-beneficial rhizobacteria in a competitive soil environment (**Figure 5B**). Interestingly, mycorrhization of maize was recently reported to boost DIMBOA production [38]. Since mycorrhization is known to cause major qualitative changes in rhizobacterial communities [39], it is possible that increased DIMBOA exudation from mycorrhizal roots contributes to this so-called mycorrhizosphere effect.

The BX content of maize roots has been studied extensively because of their demonstrable roles as allelochemicals [13], [18]. Recent studies have identified the glucosides HDMBOA-glc and DIMBOA-glc as the principal BXs in roots and root exudates of maize [16], [40]. DIMBOA was identified in both studies as only a minor component of the total root BX content. A possible explanation for this discrepancy lies in the different methods of BX extraction. In our study, entire root systems were incubated in water for 7 hours, whereas Robert et al. [40] used direct sampling with a 50% (v/v) water:methanol extraction buffer on the root surface. Hence, the latter method analysed root-exuded BXs directly, while our method assessed root-exuded BXs after prolonged incubation of the root system in water. Since BX glucosides are readily hydrolysed in water and DIMBOA is more stable than HDMBOA [41], it may not be surprising that our study identified the aglycone DIMBOA as the dominant BX from root exudates. Considering that soils constitute a hydrated environment, we propose that the more refractory DIMBOA compound functions as the long-distance BX signal, recruiting beneficial rhizobacteria.

The *P. putida* strain used in our studies was originally isolated from horticultural soil and is a competitive coloniser of rhizospheres of economically important crops [42]. Using *in vivo* expression techniques (IVET), Ramos-González *et al.* [43] identified 29 genes that are induced following 14 days of growth in the maize rhizosphere, including some with annotated functions in chemotaxis and detoxification. However, despite the similarities in general cellular functions, there were no overlapping genes between this IVET study and our transcriptome analysis. A more recent transcriptome study of *P. putida* KT2440 identified gene induction as the dominant response after 6 days of colonisation in the maize rhizosphere [44], which is in agreement with our finding that DIMBOA induces only *P. putida* gene induction. In total, Matilla *et al.* [44] revealed enhanced expression of 93 genes in the maize rhizosphere, including genes with predicted functions in general metabolism, transcriptional regulation, transport, chemotaxis and DNA metabolism. With the exception of the ISPpu14 transposase Orf1 (PP5398), there is again no overlap between this study and our transcriptome analysis. This is not surprising, since our analysis was specifically focussed on the bacterial response to DIMBOA, and not to the multitude of responses that are required for rhizopshere competence, such as attachment to the maize root surface and metabolism of the wide range of compounds besides DIMBOA in root exudates. Furthermore, the transcriptional response reported in our study was expressed within 1 hour of exposure to DIMBOA. It is, therefore, likely that these gene expression patterns are specific to the initial stages of the interaction: the bacterial response to chemical cues from the host plant in the soil before they attach and establish themselves in the rhizosphere. Since our ultimate objective was to study the maize-bacterium interaction, rather than quantitative gene expression in *P. putida* KT2440 *per se*, we made no further attempts to confirm our *in vitro* transcription profiling with a complementary technique. Therefore, it remains difficult to establish unequivocally that specific genes identified as DIMBOA-inducible *in vitro* are in fact responsible for the biological interactions described in this study. Nevertheless, it is still instructive to consider the genes in the light of what is already known about environmentally responsive *P. putida* genes. Moreover, the DIMBOA-inducible gene expression patterns associated with tolerance to *N*-heteroaromatic compounds and bacterial motility led us to conduct follow-up experiments, which revealed a novel signalling mechanism during the initial phases of the maize-*P. putida* interaction.

Motility is an essential trait for rhizosphere competence [5]. Our transcriptome analysis identified two DIMBOA-inducible genes that have been associated with bacterial chemotaxis (PP4340 and PP4888), and a third gene (PP2604) with a putative function in DIMBOA transport (**Table S1**). The DIMBOA-responsive gene *cheY* (PP4340) is a chemotactic response regulator in bacteria [45]. Furthermore, benzoate chemotaxis in *P. putida* PRS2000 depends on a methyl-accepting chemotaxis transducer (M-ACT) and an aromatic acid:H^+^ symporter (AAHS), PcaK [46]. Our transcriptome analysis identified the M-ACT homologue PP4888, and two genes, PP2241 and PP2604, belonging to the Major Facilitator Superfamily (MFS) of AAHS transporters [47]. Of the two latter genes, only PP2604 shares common features with *pcaK* (STRING v.9 database) [48]. On the basis of these motility-related transcription patterns, we considered the possibility that DIMBOA acts as a chemo-attractant for *P. putida*. This hypothesis was confirmed by our subsequent chemotaxis assays, demonstrating positive taxis of *P. putida* KT2440 towards DIMBOA (**Figure 4**).

*P. putida* KT2440 is relatively tolerant of DIMBOA in comparison to other soil bacteria (**Figure 2A**). We subsequently found that *P. putida* KT2440 accelerates degradation of DIMBOA and its direct break-down product MBOA (**Figure 2B**), indicating BX catabolism [20]. Such a mode of tolerance is supported by our transcriptome analysis, which revealed seven DIMBOA-inducible genes that may be associated with degradation of *N*-heteroaromatic compounds (**Table S1**). These genes include *nuoCD* (PP4121) and *nuoG* (PP4124), which encode subunits of NADH dehydrogenase I, PP4690 encoding a Rieske 2Fe-2S family subunit of soluble dioxygenases, PP0256 encoding a molybdopterin oxidoreductase, PP4661 encoding a putative oxidoreductase, and the α/β hydrolase-fold Superfamily genes PP4540 and PP4551, members of which catalyse degradation of the *N*-heteroaromatic compound 1*H*-3-hydroxy-4-oxoquinoline by *P. putida* 33/1 [49]. We conclude that this mechanism of BX tolerance provides *P. putida* KT2440 with a competitive advantage over other micro-organisms in exploiting the maize rhizosphere.

Our soil-based colonisation assays revealed that *P. putida* cells colonise maize roots of DIMBOA-synthesising lines in greater numbers than roots of DIMBOA-deficient lines. Although BX-dependent rhizosphere attraction of *P. putida* occurred in both autoclaved and non-autoclaved soil (**Figure 5**), the difference in *P. putida* colonisation between *BX1* and *bx1* lines in non-autoclaved soil was only consistent between both lines during relatively young developmental stages of the plants (**Figure 5B**). This age-dependent decline in *P. putida* response to BXs concurs with our finding that DIMBOA root exudation declines steadily as seedlings age (**Figure 1**). In autoclaved soil however, this age-dependence was unclear. Autoclaved soil provides a much less competitive environment for introduced *P. putida* cells than non-autoclaved soil. It is therefore possible that the lower DIMBOA exudation rates of older plants remains sufficient to attract bacteria from non-autoclaved soil to the rhizosphere. Alternatively, it is possible that HDMBOA-glc, which did not show a noticeable age-dependent decline in exudation rate (**Figure 1**), contributes to bacterial recruitment at later developmental stages of the host plant. In both autoclaved and non-autoclaved soil, numbers of other rhizosphere bacteria were similar between roots of *BX1* and *bx1* plants (**Figure 5**). The difference in response to BX-exuding roots between *P. putida* and other rhizobacteria indicates that the composition of the rhizophere microbial community is strongly influenced by the presence of DIMBOA in root exudates of the host plant. Apart from direct anti-microbial effects, DIMBOA root exudation may have an additive effect considering that DIMBOA-exposed *P. putida* showed enhanced expression of the *phzF* gene (**Table S1**), which encodes an enzyme in the biosynthesis of the broad-spectrum antibiotic phenazine [50]. Other studies have revealed bacterial attraction to primary metabolites in plant roots: L-leucine and L-malate attract *P. fluorescens* to tomato roots [51], while L-malate was found to promote attraction of *Bacillus subtilis* to the rhizosphere of *Arabidopsis thaliana* [4]. To our knowledge, DIMBOA is the first allelochemical shown to act as a chemo-attractant for beneficial rhizobacteria, and may explain why *P. putida* KT2440 is such a successful coloniser of the maize rhizosphere [29]. Our discovery also strengthens the notion that certain bacteria have acquired the ability to detoxify aromatic plant compounds, allowing them to exploit the energy-rich rhizosphere of plant roots exuding allelochemical compounds. These same bacteria can be exploited for the remediation of aromatic pollutants and herbicides [10].

In summary, our study has shown that root exudation of BXs attracts plant beneficial rhizobacteria. Although BX biosynthesis is mostly developmentally regulated [12], recent evidence has revealed that BX production by maize seedlings is to a certain extent responsive to environmental stimuli [52], [53]. It would therefore, be interesting to examine BX-dependent effects on rhizobacteria during adaptive interactions between above- and below-ground defences. Our study also provides important knowledge for agricultural programmes aiming at sustainable yield improvement of cereal crops. Management of soil-borne diseases has proved problematic, because plant roots are relatively inaccessible to fungicidal chemicals. Furthermore, growth promotion by excessive soil fertilisation can have detrimental environmental impacts. Selection of cereal varieties with increased capacity for BX root exudation may lead to crops with an improved ability to recruit disease-suppressive and growth-promoting rhizosphere communities, reducing the need for repeated applications of fungicides and fertilisers. However, there is evidence that the specialist herbivore Western Corn Rootworm (*Diabrotica virgifera*) uses root-exuded BXs, such as DIMBOA and MBOA, as feeding cues [40], [54]. The potential for crop improvement by selection for increased BX exudation should therefore be approached with caution. On the other hand, the accelerated degradation of DIMBOA and MBOA by *P. putida* (**Figure 2B**) may interfere with host location by *D. virgifera*, presenting a potential opportunity for biocontrol of this pest

## Supporting Information

Table S1 Functional annotation of *Pseudomonas putida* KT2440 genes with a statistically significant induction at 1 h after exposure to 5 µg mL^−1^ DIMBOA. Presented are 36 genes with annotated functions out of a total of 55 DIMBOA-inducible genes.
(XLSX)

## References

[1] Rangel-Castro JI, Killham K, Ostle N, Nicol GW, Anderson IC (2005). Stable isotope probing analysis of the influence of liming on root exudate utilization by soil microorganisms.. Environ Microbiol.

[2] Whipps JM (2001). Microbial interactions and biocontrol in the rhizosphere.. J Exp Bot.

[3] Haichar FZ, Marol C, Berge O, Rangel-Castro JI, Prosser JI (2008). Plant host habitat and root exudates shape soil bacterial community structure.. ISME J.

[4] Rudrappa T, Czymmek KJ, Pare PW, Bais HP (2008). Root-secreted malic acid recruits beneficial soil bacteria.. Plant Physiol.

[5] Lugtenberg BJJ, Dekkers LC (1999). What makes *Pseudomonas* bacteria rhizosphere competent?. Environ Microbiol.

[6] Sofie D, Jos V, Yaacov O (2003). Plant growth-promoting effects of Diazotrophs in the rhizosphere.. Crit Rev Plant Sci.

[7] Lugtenberg B, Kamilova F (2009). Plant-growth-promoting Rhizobacteria.. Ann Rev Microbiol.

[8] Handelsman J, Stabb EV (1996). Biocontrol of soilborne plant pathogens.. Plant Cell.

[9] Van Wees SCM, Van der Ent S, Pieterse CMJ (2008). Plant immune responses triggered by beneficial microbes.. Cur Opinion Plant.

[10] Parales RE, Harwood CS (2002). Bacterial chemotaxis to pollutants and plant-derived aromatic molecules.. Cur Opinion Microbiol.

[11] Harwood CS, Rivelli M, Ornston LN (1984). Aromatic acids are chemoattractants for *Pseudomonas putida*.. J Bacteriol.

[12] Frey M, Schullehner K, Dick R, Fiesselmann A, Gierl A (2009). Benzoxazinoid biosynthesis, a model for evolution of secondary metabolic pathways in plants.. Phytochem.

[13] Niemeyer HM (2009). Hydroxamic acids derived from 2-hydroxy-2*H*-1,4-benzoxazin-3(4*H*)-one: key defense chemicals of cereals.. J Agric Food Chem.

[14] Niemeyer HM (1988). Hydroxamic acids (4-hydroxy-1,4-benzoxazin-3-ones), defence chemicals in the gramineae.. Phytochem.

[15] Morant AV, Jorgensen K, Jorgensen C, Paquette SM, Sanchez-Perez R (2008). Beta-Glucosidases as detonators of plant chemical defense.. Phytochem.

[16] Glauser G, Marti G, Villard N, Doyen GA, Wolfender JL (2011). Induction and detoxification of maize 1,4-benzoxazin-3-ones by insect herbivores.. Plant J.

[17] Ahmad S, Veyrat N, Gordon-Weeks R, Zhang Y, Martin J (2011). Benzoxazinoid metabolites regulate innate immunity against aphids and fungi in maize.. Plant Physiol.

[18] Niemeyer HM, Perez FJ (1994). Potential of Hydroxamic Acids in the control of cereal pests, diseases, and weeds..

[19] Woodward MD, Corcuera LJ, Helgeson JP, Upper CD (1978). Decomposition of 2,4-dihydroxy-7-methoxy-2*H*-1,4-benzoxazin-3(4*H*)-one in aqueous solutions.. Plant Physiol.

[20] Kumar P, Gagliardo RW, Chilton WS (1993). Soil transformation of wheat and corn metabolites MBOA and DIM_2_BOA into aminophenoxazinones.. J Chem Ecol.

[21] Krogh SS, Mensz SJ, Nielsen ST, Mortensen AG, Christophersen C (2006). Fate of benzoxazinone allelochemicals in soil after incorporation of wheat and rye sprouts.. J Agric Food Chem.

[22] Chase WR, Nair MG, Putnam AR, Mishra SK (1991). 2,2′-oxo-1,1′-azobenzene: microbial transformation of rye (*Secale cereale* L.) allelochemical in field soils by *Acinetobacter calcoaceticus*: III.. J Chem Ecol.

[23] Friebe A, Vilich V, Hennig L, Kluge M, Sicker D (1998). Detoxification of Benzoxazolinone allelochemicals from wheat by *Gaeumannomyces graminis* var. *tritici*, *G. graminis* var. *graminis*, *G. graminis* var. *avenae*, and *Fusarium culmorum*.. Appl Environ Microbiol.

[24] Friebe A, Wieland I, Schulz M (1996). Tolerance of *Avena sativa* to the allelochemical benzoxazolinone. Degradation of BOA by root-colonizing bacteria.. J Appl Bot-Ang Botanik.

[25] Anzai K, Isono K, Okuma K, Suzuki S (1960). The new antibiotics, questiomycins A and B.. J Antibiotics.

[26] Gerber NN, Lechevalier MP (1964). Phenazines and Phenoxaziones from *Waksmania aerata* sp. nov. and *Pseudomonas iodina**.. Biochem.

[27] Gagliardo R, Chilton W (1992). Soil transformation of 2(3*H*)-benzoxazolone of rye into phytotoxic 2-amino-3*H*-phenoxazin-3-one.. J Chem Ecol.

[28] Matilla MA, Ramos JL, Bakker PAHM, Doornbos R, Badri DV (2010). *Pseudomonas putida* KT2440 causes induced systemic resistance and changes in *Arabidopsis* root exudation.. Environ Microbiol Rep.

[29] Molina L, Ramos C, Duque E, Ronchel MC, García JM (2000). Survival of *Pseudomonas putida* KT2440 in soil and in the rhizosphere of plants under greenhouse and environmental conditions.. Soil Biol Biochem.

[30] Dechesne A, Pallud C, Bertolla F, Grundmann GL (2005). Impact of the microscale distribution of a *Pseudomonas* strain introduced into soil on potential contacts with indigenous bacteria.. Appl Environ Microbiol.

[31] Nelson KE, Weinel C, Paulsen IT, Dodson RJ, Hilbert H (2002). Complete genome sequence and comparative analysis of the metabolically versatile *Pseudomonas putida* KT2440.. Environ Microbiol.

[32] Miyakoshi M, Shintani M, Terabayashi T, Kai S, Yamane H (2007). Transcriptome analysis of *Pseudomonas putida* KT2440 harboring the completely sequenced IncP-7 Plasmid pCAR1.. J Bacteriol.

[33] Dabney AR, Storey JD (2007). A new approach to intensity-dependent normalization of two-channel microarrays.. Biostatistics.

[34] Saeed AI, Sharov V, White J, Li J, Liang W (2003). TM4: A free, open-source system for microarray data management and analysis.. Biotechniques.

[35] Tusher VG, Tibshirani R, Chu G (2001). Significance analysis of microarrays applied to the ionizing radiation response.. Proc Natl Acad Sci U S A.

[36] Agrios GN (2005). Plant Pathology.

[37] Jones DL, Nguyen C, Finlay RD (2009). Carbon flow in the rhizosphere: carbon trading at the soil-root interface.. Plant Soil.

[38] Song YY, Cao M, Xie LJ, Liang XT, Zeng RS (2011). Induction of DIMBOA accumulation and systemic defense responses as a mechanism of enhanced resistance of mycorrhizal corn (*Zea mays* L.) to sheath blight.. Mycorrhiza.

[39] Linderman RG (1988). Mycorrhizal interactions with the rhizosphere microflora - the Mycorrhizosphere Effect.. Phytopathol.

[40] Robert CAM, Veyrat N, Glauser G, Marti G, Doyen GR (2012). A specialist root herbivore exploits defensive metabolites to locate nutritious tissues.. Ecol Lett.

[41] Maresh J, Zhang J, Lynn DG (2006). The innate immunity of Maize and the dynamic chemical strategies regulating Two-Component signal transduction in *Agrobacterium tumefaciens*.. Chem Biol.

[42] Nakazawa T (2002). Travels of a *Pseudomonas*, from Japan around the world.. Environ Microbiol.

[43] Ramos-Gonzalez MI, Campos MJ, Ramos JL (2005). Analysis of *Pseudomonas putida* KT2440 gene expression in the maize rhizosphere: In vitro expression technology capture and identification of root-activated promoters.. J Bacteriol.

[44] Matilla MA, Espinosa-Urgel M, Rodriguez-Herva JJ, Ramos JL, Ramos-Gonzalez MI (2007). Genomic analysis reveals the major driving forces of bacterial life in the rhizosphere.. Genome Biol.

[45] Wolanin PM, Webre DJ, Stock JB (2003). Mechanism of phosphatase activity in the chemotaxis response regulator CheY.. Biochem.

[46] Harwood CS, Nichols NN, Kim MK, Ditty JL, Parales RE (1994). Identification of the *pcaRKF* gene cluster from *Pseudomonas putida*: involvement in chemotaxis, biodegradation, and transport of 4-hydroxybenzoate.. J Bacteriol.

[47] Pao SS, Paulsen IT, Saier MH (1998). Major facilitator superfamily.. Microbiol Mol Biol Rev.

[48] Szklarczyk D, Franceschini A, Kuhn M, Simonovic M, Roth A (2011). The STRING database in 2011: functional interaction networks of proteins, globally integrated and scored.. Nucl Acids Res.

[49] Fischer F, Kunne S, Fetzner S (1999). Bacterial 2,4-dioxygenases: new members of the alpha/beta hydrolase-fold superfamily of enzymes functionally related to serine hydrolases.. J Bacteriol.

[50] Blankenfeldt W, Kuzin AP, Skarina T, Korniyenko Y, Tong L (2004). Structure and function of the phenazine biosynthetic protein PhzF from *Pseudomonas fluorescens*.. Proc Natl Acad Sci U S A.

[51] de Weert S, Vermeiren H, Mulders IHM, Kuiper I, Hendrickx N (2002). Flagella-driven chemotaxis towards exudate components is an important trait for tomato root colonization by *Pseudomonas fluorescens*.. Mol Plant-Microbe Interact.

[52] Erb M, Flors V, Karlen D, de Lange E, Planchamp C (2009). Signal signature of aboveground-induced resistance upon belowground herbivory in maize.. Plant J.

[53] Erb M, Gordon-Weeks R, Flors V, Camanes G, Turlings TC (2009). Belowground ABA boosts aboveground production of DIMBOA and primes induction of chlorogenic acid in maize.. Plant Signal Behaviour.

[54] Bjostad LB, Hibbard BE (1992). 6-Methoxy-2-benzoxazolinone: a semiochemical for host location by Western Corn Rootworm larvae.. J Chem Ecol.

